# Engineered biomaterials for cancer immunotherapy

**DOI:** 10.1002/mco2.8

**Published:** 2020-05-27

**Authors:** Lulu Cai, Jialu Xu, Zhenglin Yang, Rongsheng Tong, Ziliang Dong, Chao Wang, Kam W. Leong

**Affiliations:** ^1^ Personalized Drug Therapy Key Laboratory of Sichuan Province Department of Pharmacy Sichuan Provincial People's Hospital School of Medicine University of Electronic Science and Technology of China Chengdu China; ^2^ Institute of Functional Nano & Soft Materials (FUNSOM) Jiangsu Key Laboratory for Carbon‐based Functional Materials and Devices Soochow University Suzhou China; ^3^ Sichuan Provincial Key Laboratory for Human Disease Gene Study the Institute of Laboratory Medicine Sichuan Provincial People's Hospital School of Medicine University of Electronic Science and Technology of China Chengdu China; ^4^ Department of Biomedical Engineering Columbia University New York USA

**Keywords:** anticancer immunity, biomaterials, cancer immunotherapy, localized delivery

## Abstract

Although cancer immunotherapy is showing tremendous promise and has progressed to the clinic, it has only achieved sporadic efficacy, with only a fraction of patients benefitting from the therapy and with undesirable side effects due to poor selectivity and high doses. Localized delivery of immunomodulators to activate anticancer immunity in situ avoids overactivation of the systemic immune system and reduces side effects. Engineered biomaterials—implantable, injectable, or transdermal—fabricated into drug delivery devices are critical components for the development of localized cancer immunotherapies. In this review, we briefly summarize progress in the application of engineered biomaterials to the localized delivery of cancer immunotherapy.

## 1 INTRODUCTION

The immune system plays an important role in preventing tumor development and cancer.^1^, ^2^, ^3^, ^4^ Cancer immunotherapy employs intrinsic immune system activity to fight cancer,^5^, ^6^, ^7^, ^8^, ^9^ awakening and training the patient's own immune system to kill tumor cells.^10^, ^11^ Immunotherapy is a more powerful and safer alternative to traditional therapies such as surgery,^12^ radiotherapy,^13^ and chemotherapy and has become a standard approach for cancer treatment. To date, there have been three main types of cancer immunotherapy: immune checkpoint blockades,^14^, ^15^, ^16^ chimeric antigen receptor‐modified T cells,^17^ and cancer vaccines.^18^, ^19^, ^20^ Excellent success has been achieved using these immunotherapeutics for some tumor types, but challenges remain. Due to tumor heterogeneity,^4^ many patients do not respond—the overall objective response rate is only ∼20%.^21^, ^22^, ^23^ In addition, complications including autoimmune diseases,^24^, ^25^ nonspecific inflammation,^26^ and unexpected toxicities including cytokine release syndrome, macrophage activation syndrome, and neurotoxicity pose severe threats to patients’ lives.^27^, ^28^

Localized delivery of immunotherapeutics may improve efficacy and safety by activating anticancer immunity only where needed, reducing drug doses and avoiding overactivation of the systemic immune system.^10^, ^29^, ^30^ Engineered biomaterials—implantable,^31^ injectable,^32^, ^33^, ^34^ and transdermal materials^35^ and devices—are essential in current newly developed localized delivery systems for immune therapeutics.^36^, ^37^, ^38^, ^39^, ^40^, ^41^, ^42^, ^43^ Rationally designed biomaterials are used to optimize pharmacokinetics by improving drug accumulation, spatially controlled release, and drug retention within target locations, while reducing off‐target immunotoxicity.

**FIGURE 1:**
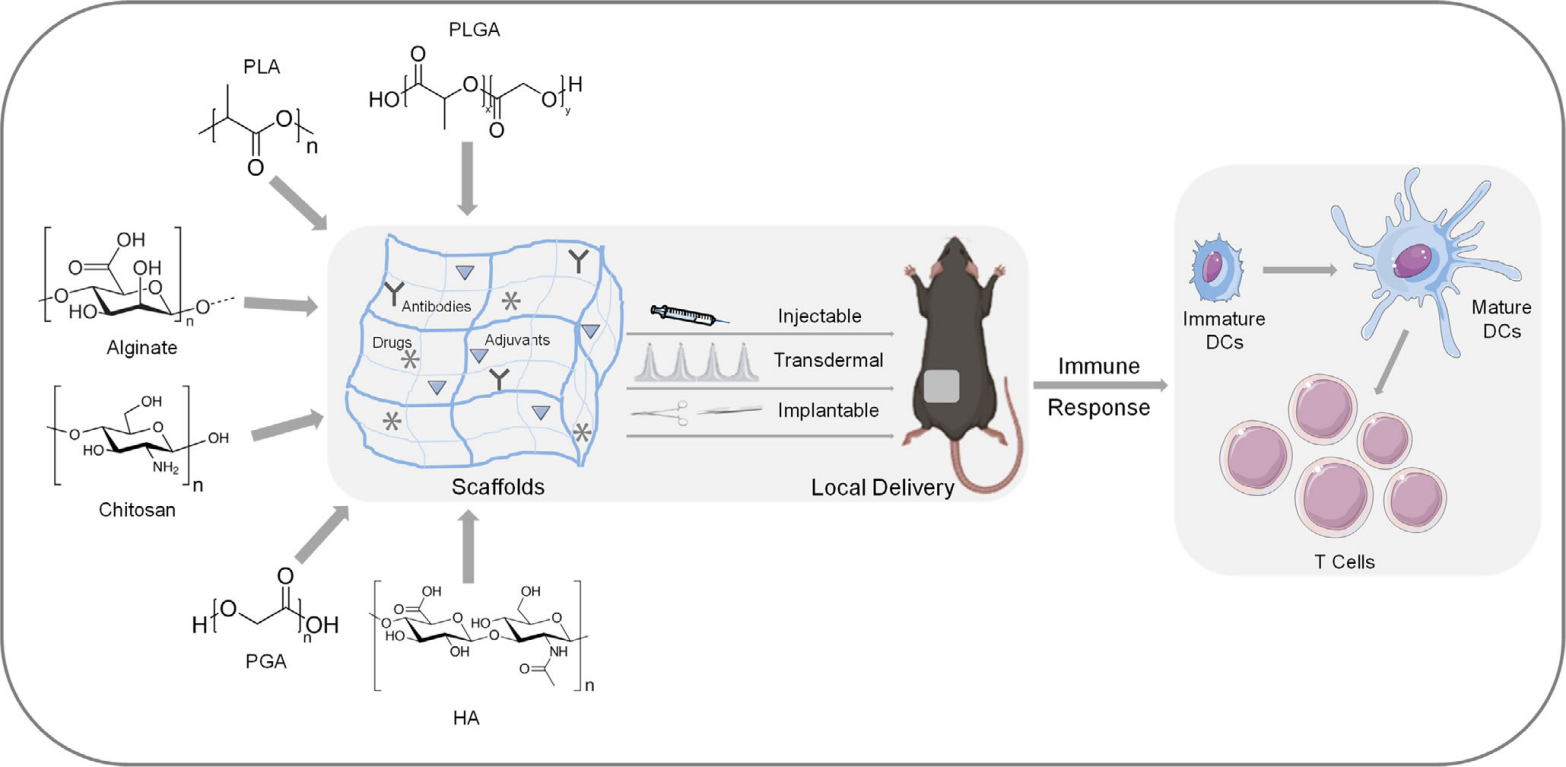
Schematic of engineered biomaterials for localized delivery of cancer immunotherapeutics. Engineered polymeric biomaterials such as polylactide (PLA), polyglycolide (PGA), poly(lactic‐co‐glycolic acid) (PLGA), hyaluronic acid (HA), alginate, and chitosan are used to create porous scaffolds that encapsulate therapeutic agents in implantable, injectable, or transdermal delivery systems. Localized delivery activates local immune responses such as maturation of dendritic cells and proliferation of T cells

The application of engineered biomaterials to cancer immunotherapy has been reviewed elsewhere.^26^, ^44^, ^45^, ^46^, ^47^, ^48^, ^49^, ^50^, ^51^ In this review, we focus on localized delivery of immunotherapy and the biomaterials—from hydrogels^52^ to microneedles^53^, ^54^—that enable this cancer treatment approach (Figure 1). It is hoped that this review will help interested nonexperts understand the latest developments, future prospects, and current challenges in the integration of engineered biomaterials and cancer immunotherapy, which may lead to new paradigms in cancer treatment.

## 2 ENGINEERED BIOMATERIALS FOR LOCALIZED DELIVERY OF IMMUNOTHERAPIES

### 2.1 Implantable biomaterials

Implantable biomaterials^55^ include biomaterials implanted subcutaneously or in place of excised tissue via minor surgery. Before implantation, the materials are functionalized or preloaded with therapeutic biological factors, chemical agents, or cells. The bioactive substances are released in a controlled manner from a large porous scaffold matrix, and immune cells are recruited to the implant site.^56^, ^57^ Examples of engineered polymeric biomaterials used as scaffolds in implantable delivery systems include poly(lactide‐co‐glycolide) (PLG),^58^, ^59^ alginate (ALG),^31^, ^60^ polyglyconate and porcine gelatin,^61^ collagen, and hyaluronic acid (HA).^62^

Dendritic cells (DCs) are antigen‐presenting cells (APCs) that play a major role in immune induction.^63^ DCs cause humoral and cellular immune responses by presenting tumor‐associated antigens in different ways. Mouse cancer models have shown that DCs capture antigens from live or dead tumor cells and present the antigens to T cells in tumor‐draining lymph nodes (the first organs of metastasis in malignant melanomas and most carcinomas), leading to production of tumor‐specific cytotoxic T lymphocytes (CTLs).^64^, ^65^, ^66^ DC‐based cancer vaccines are one type of cancer immunotherapy, and involve activating a patient's DCs or DC precursors ex vivo before returning them to the patient's body.^67^, ^68^ The activated mature DCs home to lymph nodes and present tumor antigens to naïve T cells, resulting in tumor‐specific T‐cell activation and proliferation and an antitumor response.^69^ However, there are several major issues with this approach: the cost is very high due to the complex procedures; the survival rate of the implanted DCs is low; and only a small fraction of the DCs home to the lymph nodes.^70^

Implantable drug‐delivery scaffolds using degradable polymers such as PLG have been used to address these issues. PLG has several advantages for use in cancer immunotherapy, including easy surface modification, high biocompatibility, customizable biodegradation rate, FDA approval for clinical use, and extensive previous use in other biomedical fields.^71^ A method was developed to prepare uniform PLG scaffolds: a 120 kD 85:15 copolymer of d,l‐lactide and glycolide was used to generate macroporous PLG matrices using a gas‐foaming process.^72^ PLG scaffolds have been applied to encapsulating granulocyte‐macrophage colony‐stimulating factor (GM‐CSF), a hematopoietic cytokine that induces expansion of macrophages, DCs, and neutrophils,^73^ inhibits cancer cell proliferation via immune‐independent effects,^74^ and indirectly regulates T‐cell activation and proliferation.^75^, ^76^ GM‐CSF attracts DCs that engulf apoptotic tumor cells. GM‐CSF released from the scaffold can also induce DC maturation.^77^, ^78^ The mature DCs then migrate to draining lymph nodes and present tumor antigens to T cells, resulting in T‐cell activation and expansion, enhancing the antitumor immune response.^79^ Roughly the same number of DCs can be recruited by delivery of GM‐CSF using PLG scaffolds as are administered in DC‐based cancer vaccines ex vivo.^20^, ^80^ The implantable GM‐CSF delivery system can achieve a longer immune response than traditional cancer vaccines by releasing GM‐CSF continuously for 2 weeks.^81^ CpG oligodeoxynucleotides (CpG‐ODNs; natural immunostimulatory adjuvants derived from bacteria and recognized by Toll‐like receptor 9^82^) have been encapsulated together with GM‐CSF in PLG scaffolds. The survival rate in a melanoma mouse model increased significantly after implanting PLG scaffolds encapsulating GM‐CSF, CpG‐ODNs, and tumor‐associated antigen, demonstrating the promise of using PLG scaffolds in DC‐based cancer vaccines.

Mesoporous silica is another engineered biomaterial used as a scaffold for localized delivery of immunotherapy. Mesoporous silica has good biocompatibility, high surface area, and a high loading capacity for a variety of agents due to its porous structure.^83^, ^84^, ^85^ To assess whether mesoporous silica was suitable for a DC‐based vaccine, Choi et al^86^ created porous scaffolds using the ordered mesoporous silica SBA‐15, and evaluated whether SBA‐15 scaffolds loaded with GM‐CSF recruited DCs in mice. GM‐CSF‐loaded scaffolds displayed continuous release of GM‐CSF over 7 days, resulting in 20% greater recruitment of DCs than a blank SBA‐15 scaffold, and greater antitumor activity.

As described above, implantable delivery systems are promising vehicles for localized cancer immunotherapy. In DC‐based cancer vaccines, implantable biomaterials can be designed to control the release of immune cell recruitment factors. Immature DCs recruited to the implant site infiltrate the scaffolds and present tumor antigens and proinflammatory signals such as pattern recognition receptor ligand adjuvants. Mature antigen‐bearing DCs migrate out of the scaffold to draining lymph nodes where they activate T cells, further promoting an antitumor immune response.^87^ Implantable scaffolds are advantageous due to their ability to continuously release bioactive factors for a sustained period. One disadvantage of implantable delivery systems versus other approaches described below is the requirement for surgery, which involves foreign‐body wound healing and the potential for infection.

### 2.2 Injectable biomaterials

Injectable delivery systems use hydrogels, cryogels, inorganic scaffolds, or other materials to enable the localized and controlled release of cancer therapeutics.^88^, ^89^, ^90^, ^91^ Injectable systems avoid the surgery‐related drawbacks of implantable systems and are especially well‐suited for combination therapy with chemotherapeutics or radioisotope therapy (RIT). This section will focus on hydrogel‐based injectable delivery systems.

Hydrogels are water‐like networks that mimic the physical cross‐linking of natural extracellular matrix, and are formed by minimally invasive injection of stimulus‐reactive copolymers. Biomaterials used in hydrogel‐based injectable delivery systems must have the following properties: good biocompatibility, degradability, liquid or sol‐like state at room temperature, and conversion to gel form at body temperature. These requirements are more sophisticated than those for implantable biomaterials and are more difficult to achieve.

Injectable hydrogels have been applied to developing cancer vaccines. Lee and coworkers^92^ designed an injectable smart hydrogel that self‐assembles into a microporous network similar to extracellular matrix, and can be injected subcutaneously using simple hypodermic needles. The temperature‐responsive copolymer poly(ε–caprolactone–co–lactide)–b–poly(ethyleneglycol)–b–poly(ε‐caprolactone–co‐lactide) (PCLA) was combined with HA to construct a scaffold for encapsulating GM‐CSF and ovalbumin (OVA, a model antigen). A single injection of this loaded hydrogel into B16/OVA mice led to improved recruitment of DCs and other immune cells to the tumor site and significant inhibition of tumor growth. Sung and coworkers^93^ designed a bioinspired catechol (CA)‐functionalized HA hydrogel system consisting of positively charged N‐trimethyl chitosan (TMC) and negatively charged poly(γ‐glutamic acid) (γ‐PGA) to encapsulate OVA. Chitosan has been used previously as an adjuvant to stimulate APCs, and γ‐PGA has been used to form stable nanoparticles by self‐assembly, a useful attribute for an injectable delivery system. After a single subcutaneous injection of the loaded hydrogel, the nanoparticles were continuously released and were engulfed by APCs, triggering an antigen‐specific antibody response; OVA was also taken up by APCs to induce a long‐term immune memory response.

Injectable hydrogels have shown tumor growth inhibition and prevention of recurrence in combination therapy in vivo. Wang and coworkers^94^ used a fibrin hydrogel with excellent biodegradability and biocompatibility to load the chemotherapeutics cyclophosphamide (CTX; a small molecule drug) and anti‐PD‐L1 (an immune checkpoint‐blocking monoclonal antibody). Due to the large difference in size between CTX and anti‐PD‐L1, their drug release kinetics varies widely. The smaller molecule, CTX, is released first from the fibrin gel and generates an immunogenic tumor microenvironment phenotype, which maximizes the efficacy of the more slowly released anti‐PD‐L1. This staggered delivery of the small molecule drug and the immune checking‐blocking antibody from the fibrin gel reservoir has a synergistic antitumor effect. This novel fibrin gel system was evaluated in mouse models of TNBC 4T1 breast cancer and ID8 ovarian cancer, and showed inhibition of tumor recurrence after surgery.

**FIGURE 2:**
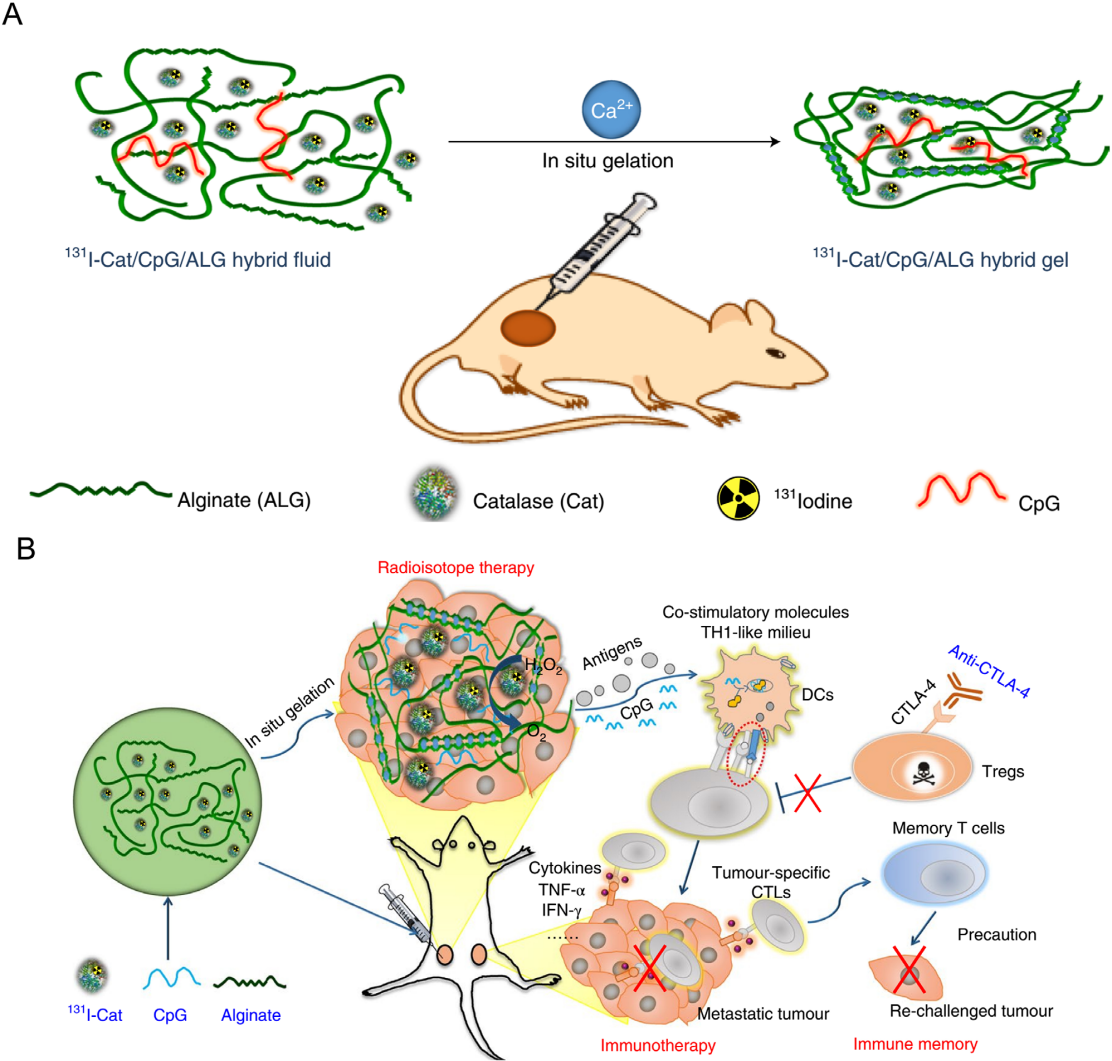
Injectable hydrogels in combination radioisotope therapy (RIT) and immunotherapy. A, Schematic of gelation of 131I‐Cat/ALG hybrid fluid upon injection into a tumor. B, Antitumor immune response induced by combination treatment with 131I‐Cat/CpG/ALG and an immune checkpoint‐blocking antibody^95^ (Copyright 2018, Springer Nature)

Injectable hydrogels have been used in combination cancer therapy involving RIT and immunotherapy. Liu and coworkers^95^ constructed a hydrogel system composed of biocompatible sodium ALG, a water‐soluble natural polysaccharide that rapidly forms a hydrogel at physiological Ca^2+^ concentrations upon injection (Figure 2). ALG was loaded with catalase (Cat), CpG, and ^131^iodine to generate a delivery system that forms a gel in situ. CpG is an oligonucleotide that acts as an immune adjuvant, contributing to a systemic antitumor immune response and enhancing the immunity of tumor antigens after RIT. CTLA‐4 checkpoint inhibitors were also incorporated in the combination therapy, resulting in significant systemic antitumor responses and effective inhibition of primary and distant tumor growth in three animal models: 4T1 murine breast cancer tumors, human prostate cancer patient‐derived xenografts (PDX) grown in mice, and rabbit VX_2_ liver cancer tumors. This ALG hydrogel‐based combination therapy has the potential for clinical translation due to its low toxicity and high efficacy, and offers a novel strategy for combining immunotherapy and radiotherapy.

Chen et al^96^ developed a different approach: a hydrogel spray applied to the surface of the tumor site following tumor resection surgery that inhibits tumor regrowth by increasing the pH in the tumor microenvironment. For the spray, the group chose an FDA‐approved fibrin gel to encapsulate CaCO_3_ nanoparticles loaded with anti‐CD47 antibody. The CaCO_3_ nanoparticles in the fibrin gel served not only as a reservoir for releasing the immunomodulatory antibody but also as a proton scavenger that reduced the acidity (increased the pH) of the tumor environment. When sprayed onto the tumor site following resection, the CaCO_3_ nanoparticles gradually dissolved and released the encapsulated anti‐CD47 antibody, which in turn activated macrophages to engulf cancer cells, and inhibited tumor recurrence at the resection site and at distant sites in a B16F10 mouse model.

**FIGURE 3:**
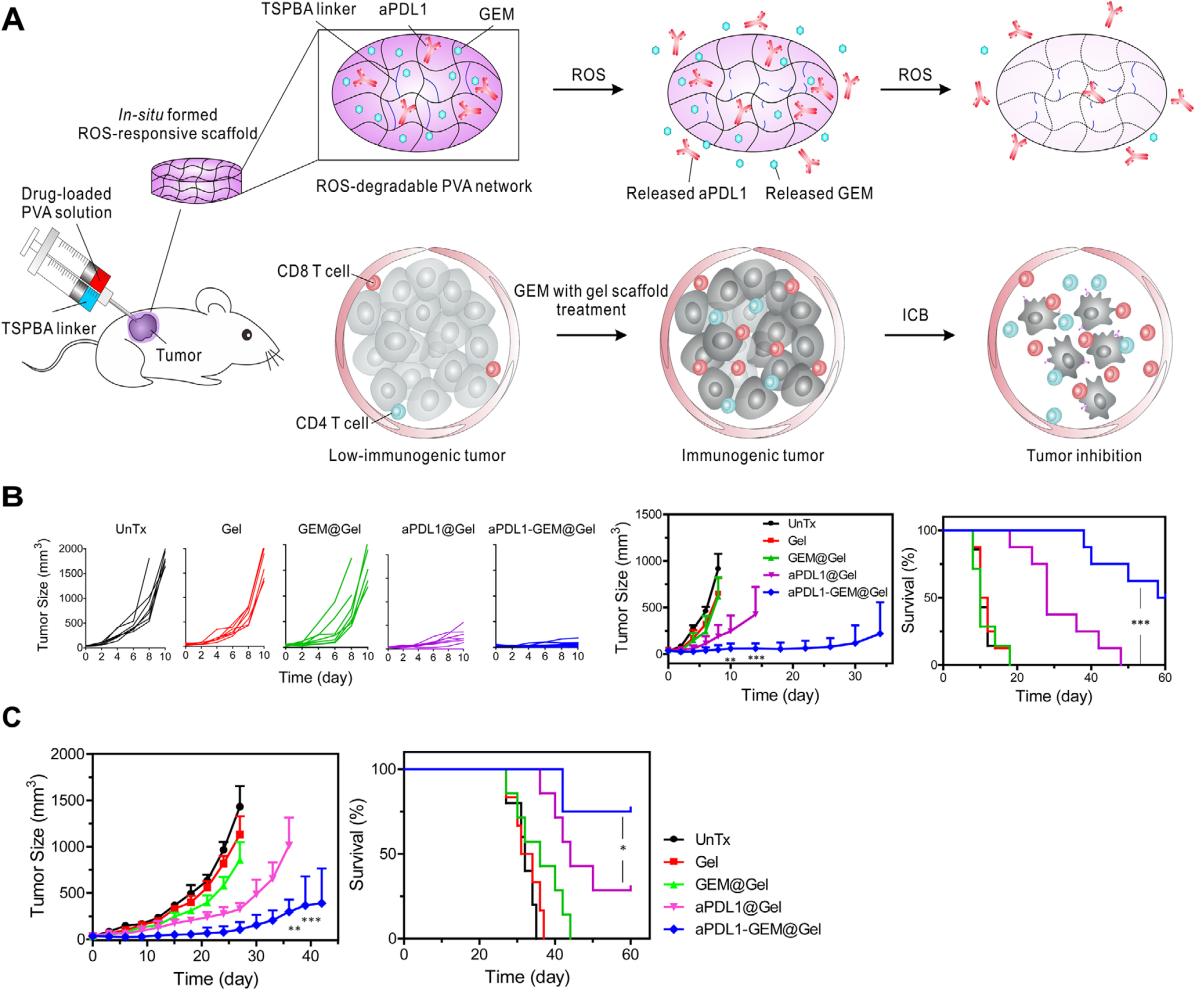
Injectable hydrogel for localized delivery of chemotherapy and immunotherapy. A, Schematic of localized delivery of anti‐PD‐L1 and gemcitabine (GEM) using an ROS‐degradable injectable hydrogel. B, SEM image of the hydrogel. Scale bar: 0.5 μm. Inset (zoomed‐in) scale bar: 0.1 μm. C‐D, Tumor growth inhibition in a B16F10 melanoma mouse model. E, Tumor growth inhibition in a 4T1 breast tumor model^97^ . GEM: Gemcitabine, ROS: Reactive oxygen species, ICB: Immune Checkpoint Blockade. (Copyright 2018, American Association for the Advancement of Science)

Elevated reactive oxygen species (ROS) in the tumor microenvironment are associated with tumor‐induced immunosuppression. Therefore, one strategy to reduce immunosuppression has been used to reduce the level of ROS during combination therapy. Wang et al^97^ generated an ROS‐responsive injectable hydrogel to deliver anti‐PD‐L1 and the chemotherapeutic gemcitabine, and reduced the ROS within the tumor. The injectable hydrogel was formed by crosslinking poly(vinyl alcohol) (PVA) and TSPBA (an ROS‐labile linker) (Figure 3). The PVA‐TSPBA polymer formed a hydrogel upon injection into mice, and the combined chemotherapy and immunotherapy enhanced the immune response and inhibited tumor growth and recurrence in B16F10 melanoma and 4T1 breast tumor models. Injectable biomaterials can be used to deliver a variety of agents that alter the tumor microenvironment to inhibit tumor growth by reducing immunosuppression, creating a favorable immune niche, and reversing various factors such as the pH and ROS level of the tumor microenvironment.

As described above, injectable biomaterials provide a surgery‐free approach to achieve sustained, localized delivery of cancer immunotherapeutics. Upon minimally invasive injection using a needle or catheter, injectable biomaterials undergo a phase transition from liquid to gel, forming biodegradable depots with tunable controlled release properties. There are several challenges in translating injectable biomaterials: First, deposition in deep tissues poses risks despite advances in image‐guided injections. Second, the in situ gelation process may prove difficult to control the subsequent release kinetics of the entrapped therapeutic cargo; too fast a gelation would render the injection difficult, too slow would see the cargo leaches out before the depot is formed. A possible solution would be to build in associations between the drug and the scaffolding biomaterials, either ionic or covalent, so that the release kinetics would be determined by the cleavage of the drug‐biomaterial bond and not the gelation kinetics. Third, the material requirements for injectable biomaterials, including considerations of biocompatibility and liquid‐to‐gel phase transition kinetics, add another level of complexity for clinical translation.

Although implantable biomaterials and injectable biomaterials have more or less small defects, in general, they still have specific advantages. For example, implantable biomaterials can prolong the release time of the agents. By using degradable biomaterials, there is no need for secondary surgical removal. After the degradation of the biomaterials, the tissue in the implanted area can be regenerated, and the implant does not interfere with radiography. Similarly, injectable biomaterials have unique strength, such as convenient formulation, shape fitness to body cavities, ease of depot size control, and sustained local release of agents. In view of these advantages, they have been extensively studied to achieve true clinical application.

### 2.3 Transdermal biomaterials

The primary function of the skin is to protect the body from the environment, but it also serves as an immune surveillance system.^98^ The skin consists of three layers: the stratum corneum (outermost layer), the epidermis (middle layer), and the dermis (inner layer).^99^ Transdermal delivery—delivery through the skin—is often clinically superior to injections or surgically implanted devices and avoids the hepatic first‐pass extraction that occurs in oral drug administration. In transdermal delivery, drugs are transported from a patch through the skin and into the systemic circulation at a pharmacologically relevant rate.^100^, ^101^ One issue with transdermal delivery is low efficiency of drug delivery due to the low permeability of the stratum corneum. Approaches used to address this low permeability include chemical enhancers,^102^ lipid enhancers,^103^ electric fields (iontophoresis and electroporation),^104^ and pressure waves generated by ultrasound or photoacoustic effects.^105^, ^106^ Another approach is to use microneedles that puncture and permeabilize the skin.^107^, ^108^, ^109^, ^110^ Microneedles are arrays containing hundreds of micrometer‐long solid needles applied using patches to create micrometer‐scale pathways through the skin for drug delivery. Due to the microscopic size of the needles and the lack of nerves in the stratum corneum, this approach is nearly painless and is effective for delivering drugs directly to the upper epidermis or dermis.^111^ A variety of materials have been used for production of microneedles, including metals, polymers, glass, and ceramics. Several varieties of microneedles exist, including solid, layered, and dissolving microneedles (the most often‐used form). Microneedles have been applied to the development of transdermal delivery systems for drugs, proteins, genetic materials, and vaccines.^112^, ^113^, ^114^, ^115^

**FIGURE 4:**
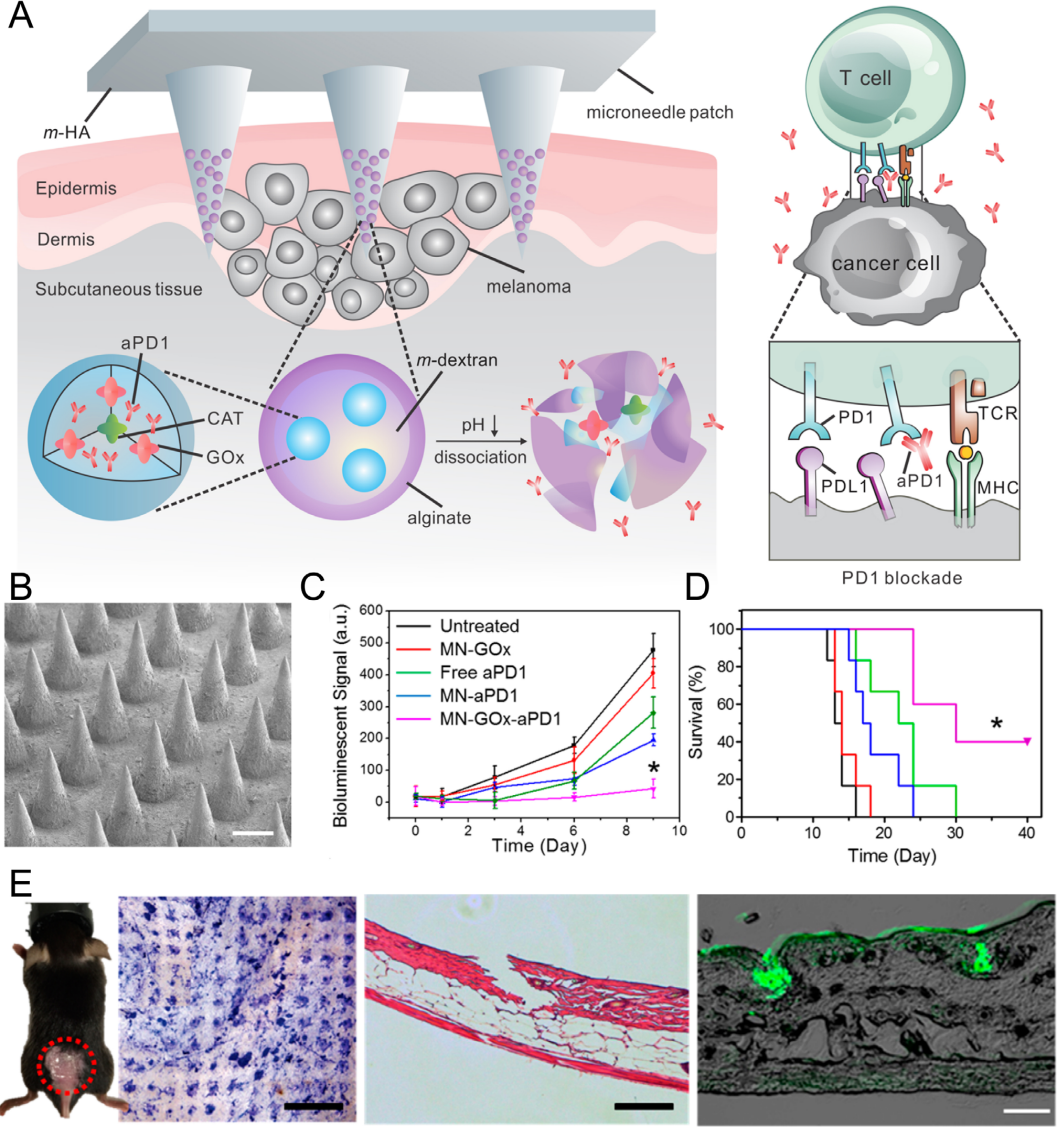
Transdermal delivery of drug‐loaded, pH‐sensitive dextran nanoparticles using microneedles for melanoma treatment. A, Schematic of microneedle delivery of anti‐PD‐1 (aPD1)‐loaded nanoparticles and pH‐induced drug release. B, SEM image of microneedles (scale bar, 200 μm). C, Tumor growth. D, Kaplan‐Meier survival curves for treated and control mice (eight mice per group). E, Image of penetration of microneedle into mouse skin (left), H&E stain (middle), and image of mouse skin penetrated by FITC‐antibody‐loaded microneedles (right)^36^. PD‐1: Programmed cell death‐1, PD‐L1: Programmed death‐ligand 1, CAT: Catalase, GOx: glucose oxidase enzyme, TCR: T‐cell receptor, MHC: Major histocompatibility complex.(Copyright 2016, American Chemical Society)

Melanoma is a serious form of skin cancer and the most common malignancy.^116^ Cancer immunotherapy has been widely researched for melanoma treatment, and the systemic administration of immune checkpoint inhibitors has shown excellent results in clinical trials.^117^, ^118^, ^119^ However, systemic cancer immunotherapy is costly and serious side effects cannot be avoided.^120^, ^121^ To reduce side effects inherent in systemic delivery, Wang et al designed microneedles for localized delivery of immune checkpoint inhibitors and other agents. HA, anti‐PD‐1, and glucose oxidase (GOx) were encapsulated within pH‐sensitive dextran nanoparticles for melanoma treatment via transdermal delivery using microneedles (Figure 4).^36^ In the tumor microenvironment, the microneedles dissolved and the porous nanoparticles released anti‐PD‐1 continuously. In a B16F10 melanoma mouse model, the survival rate was greater when using microneedles than when using intratumor (*i.t*.) or intravenous (*i.v*.) injections of the same dose of anti‐PD‐1. When anti‐PD‐1 was co‐delivered with anti‐CTLA4 (another checkpoint antibody), tumor growth was suppressed and antitumor efficacy improved. In a different study, the natural biological pigment melanin was encapsulated along with whole tumor lysate within a microneedle patch. When the microneedle patch was applied to the skin, the microneedles degraded and released tumor lysate, activating an immune response. When exposed to near‐infrared irradiation, the heat generating by the melanin caused local release of inflammatory cytokines, further activating the immune response. When applied to the B16F10 model, this transdermal delivery system produced a strong innate and adaptive immune response and induced tumor regression. These results demonstrated that the anticancer vaccine could be delivered using microneedles to improve the survival rate in a melanoma mouse model.^122^ Ye et al^123^ developed a highly drug‐concentrated hybrid core‐shell microneedle system to deliver the immune checkpoint inhibitors anti‐PD‐L1 and 1‐methyl‐D,L‐tryptophan (1‐MT) to treat melanoma. The microneedle system enriched the drugs at the tip of the microneedles through electrostatic interactions, improving transdermal delivery. The use of PVA increased the loading of 1‐MT in the microneedles and prevented crystallization of the agents. In vivo and in vitro results showed high transdermal drug delivery efficiency, and the residence time of anti‐PD‐L1 was as long as 2 days. In a B16 melanoma mice model, microneedles had significantly greater tumor growth inhibition than intratumoral injection, possibly due to improved recruitment of T lymphocytes. This core‐shell microneedle system provides a promising new platform for localized co‐delivery of antibodies and chemotherapeutics.

As described above, microneedles can be degradable or removable and can be used in transdermal delivery of antibodies, vaccines, and other therapeutic agents for treatment of tumor. Microneedle‐based transdermal delivery of cancer immunotherapeutics has shown promise in vivo and is poised for translation to large‐scale clinical use.

## 3 CONCLUSIONS

Enhancing or restoring the immune system's ability to recognize and destroy malignant cells is now a standard approach to cancer treatment. Current cancer immunotherapies such as immune checkpoint blockade and cancer vaccines have attracted great attention due to their successes, but still pose significant challenges due to their sporadic efficacy—many patients do not benefit from the therapy. To improve treatment efficacy, cancer immunotherapy has been combined with other strategies such as chemotherapy and radiation therapy, with the goal of achieving synergistic treatment. However, these systemic combination therapies typically suffer from severe side effects. Localized drug delivery addresses the problem of toxicity and side effects, and one key to successful localized delivery is choosing the right engineered biomaterials to act as reservoirs or drug carriers. As described in this review (Table 1), engineered biomaterials are used as scaffolds in implantable, injectable, or transdermal delivery devices to allow tunable drug release kinetics and delivery for up to weeks, and lower drug doses, improving the efficacy and safety of cancer immunotherapy. These biomaterial scaffolds can be loaded with chemical agents, cells, tumor‐associated antigens, and/or adjuvants that directly activate the immune system or that modular the tumor microenvironment.

**TABLE 1:** Summary of representative engineering biomaterials in this review

Delivery system	Biomaterial composition	Cargo	Tumor model	References
Implantable scaffold	PLG	GM‐CSF/CpG	B16F10 melanoma	20, 58, 59
	Mesoporous silica	GM‐CSF	C57BL/6 mice	83, 84, 85, 86
Injectable/spreadable hydrogel	PCL/HA	GM‐CSF/OVA	B16‐OVA melanoma	92
	TMC/γ‐PGA/HA	OVA	C57BL/6 mice	93
	fibrin	CTX/anti‐PD‐L1	TNBC 4T1 breast cancer; ID8 ovarian cancer	94
	ALG	Cat/CpG/^131^I	4T1 murine breast cancer tumors; PDX grown in mice; rabbit VX_2_ liver tumors	95
	Fibrin	CaCO_3_/ anti‐CD47	B16F10 melanoma	96
Transdermal microneedle	HA	anti‐PD‐1/GOx	B16F10 melanoma	36, 124
	PVA	anti‐PD‐L1/1‐MT	B16 melanoma	123, 125

PLG: poly(lactic‐co‐glycolic acid), GM‐CSF: Granulocyte‐macrophage colony‐stimulating factor, PCL: Polycaprolactone, HA: Hyaluronic acid, TMC: trimethylene carbonate, γ‐PGA: Poly‐γ‐glutamic acid, ALG: alginate, PVA: Poly(vinyl alcohol)

These innovative delivery systems have great potential for improving cancer immunotherapy, but several limitations must be considered. Because biomaterials are foreign to the body, they may cause an acute inflammatory response. In addition, chronic inflammation may occur during degradation of biomaterials. Clinical applications of all types of cancer immunotherapy are limited by their cost and by the difficulty of isolating cells from patient, and expanding and engineering the cells before re‐administration. The approval of biomaterials by the FDA is necessary for expedited clinical translation, but many biomaterials are not FDA approved. Great efforts are still needed to bring localized delivery of cancer immunotherapy using engineered biomaterials to the clinic.
